# Relative Risk Chart Score for the Assessment of the Cardiovascular Risk in Young Patients with Ankylosing Spondylitis

**DOI:** 10.1155/2018/1847894

**Published:** 2018-02-15

**Authors:** Javier Rueda-Gotor, Fernanda Genre, Alfonso Corrales, Ricardo Blanco, Patricia Fuentevilla, Virginia Portilla, Rosa Expósito, Cristina Mata Arnaiz, Trinitario Pina, Carlos González-Juanatey, Luis Rodriguez-Rodriguez, Miguel A. González-Gay

**Affiliations:** ^1^Epidemiology, Genetics and Atherosclerosis Research Group on Systemic Inflammatory Diseases, Division of Rheumatology, Hospital Universitario Marqués de Valdecilla, IDIVAL, University of Cantabria, Santander, Spain; ^2^Division of Rheumatology, Hospital Comarcal, Laredo, Cantabria, Spain; ^3^Division of Cardiology, Hospital Lucus Augusti, Lugo, Spain; ^4^Division of Rheumatology, Instituto de Investigación Sanitaria del Hospital Clínico San Carlos (IDISSC), Hospital Clínico San Carlos, Madrid, Spain

## Abstract

**Objective:**

To determine if the use of the relative risk (RR) chart score may help to identify young ankylosing spondylitis (AS) patients at high risk of cardiovascular (CV) disease.

**Methods:**

73 AS patients younger than 50 years were assessed. CV risk was calculated according to the total cholesterol systematic coronary risk evaluation (TC-SCORE) and the RR chart score. C-reactive protein (CRP) value at disease diagnosis and carotid ultrasound data were also analyzed.

**Results:**

Twenty (27.4%) patients exhibited carotid plaques being classified into the category of very high CV risk. None of them was found to have a high/very high TC-SCORE. CRP > 3 mg/L at disease diagnosis was associated with the presence of carotid plaques (odds ratio 5.66, *p* = 0.03). Whereas only 5 (14.2%) of the 35 patients with RR = 1 had carotid plaques, 15 (39.5%) of 38 with RR > 1 showed plaques. A model that included the performance of carotid US in patients with RR > 1 who had CRP > 3 mg/L allowed us to identify 60% of very high risk patients, with a specificity of 77.4%.

**Conclusions:**

RR chart score assessment may help to identify young AS patients at high risk of CV disease.

## 1. Introduction

Ankylosing spondylitis (AS) is a type of inflammatory arthritis that causes low back pain in young individuals, starting typically before the age of 45 [1]. This chronic inflammatory disease is associated with an increased cardiovascular (CV) mortality [2].

The 2016 European Society of Cardiology (ESC) guidelines on CV disease prevention recommend using the systematic coronary risk evaluation (SCORE), as predictive model to estimate the individual's absolute risk for fatal CV event [3]. The SCORE includes the following variables: age, sex, lipid levels, smoking, and blood pressure [3]. The age is the variable with the greatest influence in this model [3]. Unfortunately, the SCORE has important limitations to identify high CV risk patients when applied to individuals under 50 years [3], thus precluding them from the initiation of statin therapy for primary prevention. This fact constitutes a major point of concern in AS, a condition associated with early atherosclerotic disease [4]. In this regard, we have recently reported that the SCORE underestimates the actual CV risk of patients with AS [5]. Because of that we proposed the use of additional tools, such as the use of carotid ultrasound (US) to better identify AS at high risk of CV disease [5, 6].

In an attempt to compensate for the underestimation of the CV risk in young individuals when the SCORE was applied, the 2016 ESC guidelines proposed use of a relative risk (RR) chart score instead of the traditional SCORE system in individuals younger than 50 years [3]. Unlike the SCORE, the RR does not define a 10-year absolute risk of a fatal CV event but indicates the probability of developing a fatal CV event in an individual with traditional CV risk factors (smoking, hypertension, and hypercholesterolemia) compared to another individual without them, in whom the RR would be equal to 1. According to the ESC guidelines, the RR chart score can help to identify young patients with traditional CV risk factors who have a low SCORE, promoting in these cases a stricter preventive CV management [3].

As discussed in the 2016 ESC guidelines, imaging techniques such as carotid US, are useful to improve the CV risk estimation in the general population and also in patients with AS [3, 5, 6]. Data from the general population also indicate that several biomarkers, in particular the C-reactive protein (CRP), are independent risk factors for CV disease that may help to improve CV risk stratification [3, 7].

Taking all these considerations together, in the present study we aimed to determine if the RR chart score may improve the identification of young AS patients at high risk of CV disease.

Since an increased frequency of subclinical atherosclerosis assessed by carotid US was found in AS without clinically evident CV disease [4, 8] and a relationship between the CRP levels at the time of disease diagnosis and the presence of subclinical atherosclerosis was also disclosed in these patients [8], we assessed whether both the carotid US and the CRP at the time of disease diagnosis may improve the sensitivity to identify high CV risk among young AS patients in whom the RR charts was applied.

## 2. Methods

### 2.1. Patients

A set of 73 consecutive patients between 35 and 50 years seen over a 4-year period at Hospital Universitario Marqués de Valdecilla and Hospital de Laredo (Cantabria, Northern Spain) that fulfilled definitions for AS according to the 1984 modified New York criteria [1] were recruited. Patients with history of CV events (ischemic heart disease, cerebrovascular accident, peripheral arterial disease, or heart failure), diabetes mellitus, and chronic kidney were excluded.

CV risk was estimated according to the total cholesterol- (TC-) SCORE chart designed for low-risk population such as the Spanish population and to the RR chart score, both included in the 2016 ESC guidelines [3]. The TC-SCORE is calculated considering total cholesterol [3]. Subjects with SCORE < 1% are included in the category of low risk. Those with a SCORE ≥ 1% and <5% are in the category of moderate risk. When the chart SCORE result is ≥5% and <10% they are classified as having high risk. Finally, those patients with SCORE results ≥ 10% are included in the category of very high CV risk [3].

**Figure 1:**
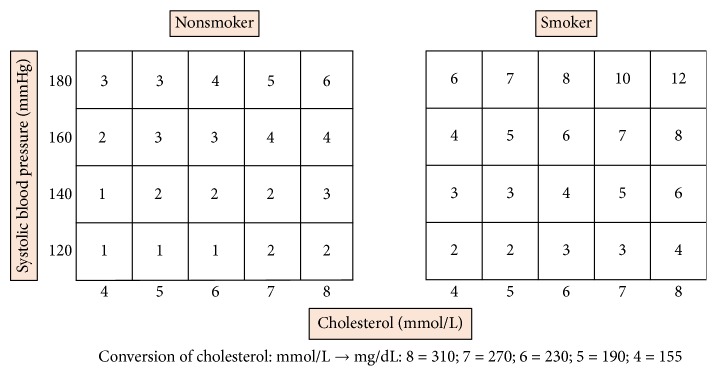
Relative risk chart, derived from SCORE. Based on the ESC 2016 guidelines [3].

The RR for a CV event was also calculated in all these AS patients considering information related to smoking, systolic blood pressure, and total cholesterol according to a RR chart score included in the 2016 ESC guidelines (Figure 1) [3]. The RR compares the absolute risk of the patient with that of another patient of the same age and sex, with ideal levels of risk factors.

Carotid US was performed as previously described [9, 10]. Briefly, carotid US examination included the measurement of carotid intima media thickness (cIMT) in the common carotid artery and the detection of focal plaques in the extracranial carotid tree. Plaque was defined as a focal protrusion in the lumen at least cIMT > 1.5 mm, protrusion at least 50% greater than the surrounding cIMT, or arterial lumen encroaching > 0.5 mm [9]. The cIMT was determined as the average of three measurements in each common carotid artery. The final cIMT was the largest average cIMT (left or right) [10]. Carotid US was performed using a commercially available scanner, Mylab 70, Esaote (Genoa, Italy) equipped with 7–12 MHz linear transducer and the automated software guided technique radiofrequency-quality intima media thickness in real-time (QIMT, Esaote, Maastricht, Holland). Patients with carotid plaques were considered as having very high CV risk [3, 10].

C-reactive protein (CRP) data at time of the disease diagnosis were also assessed. As previously described [11], a value of CRP higher than 3 mg/L was considered as a predictive factor for future CV events.

A subject's written consent was obtained in all the cases. The study was approved by the local Ethical Committee.

### 2.2. Statistical Methods

Categorical variables were described as percentages and quantitative variables as mean ± standard deviation (SD) or median (interquartile range [IQR]).

For each CV risk model, sensitivity, specificity, percentage of correctly classified patients, and area under the Receiver Operating Characteristic (ROC) curve (with 95% confidence interval) were estimated. A logistic regression model was performed to estimate the relationship between the CRP higher than 3 mg/L at disease diagnosis and presence of carotid plaques, adjusting the results for potential confounding factors (sex, age at study, arterial hypertension, dyslipidemia, obesity, and smoking).

## 3. Results

The main features of the 73 AS patients under 50 years are summarized in Table 1.

Twenty AS patients (27.4%) had carotid plaques. Interestingly a CRP value greater than 3 mg/L at disease diagnosis was associated with the presence of carotid plaques after adjusting for age, sex, obesity, hypertension, dyslipidemia, and smoking (odds ratio 5.56, 95% confidence interval 1.11–28.77; *p* = 0.03).

### 3.1. TC-SCORE Risk Calculation

Most young AS patients (*n* = 59, 80.8%) were included in the category of low CV risk when the TC-SCORE was applied. Only 14 (19.2%) were found to have moderate CV risk according to the TC-SCORE and none of them had a TC-SCORE ≥ 5%, which indicates high or very high CV risk.

Twelve of the 59 (20.3%) patients categorized as having low CV risk according to the TC-SCORE risk charts were found to have carotid plaques. The frequency of subclinical atherosclerosis was higher in those classified as having moderate CV risk since 8 (57.1%) of 14 patients with moderate CV risk according to the TC-SCORE algorithm were found to have carotid plaques.

### 3.2. Relative Risk and Carotid US Findings in Patients with AS

Only 5 of the 35 (14.2%) with RR = 1 had carotid plaques. The frequency of carotid plaques was higher in those with RR > 1 (15 [39.5%] of 38 with RR > 1) (Table 2).

### 3.3. Predictive Model to Establish the Presence of High or Very High CV Risk in Young Patients with AS

We set up a predictive model to identify young AS patients at high CV risk (Table 3).

Based on 2016 ESC guidelines, we classified patients as having high/very high CV risk if they had a TC-SCORE ≥ 5 or carotid plaques. Following this approach 20 of 73 patients fulfilled definitions of high/very high CV risk, all of them due to the presence of carotid plaques (model 2) since none of the 73 AS patients had a TC-SCORE ≥ 5 (model 1).

A predictive model that included patients with TC-SCORE ≥ 5% or patients with TC-SCORE ≥ 1% <5% plus carotid plaques only identified 8 (40%) of the high/very high CV risk AS patients with a specificity of 88.7% (area under the curve [AUC]: 0.64) (model 1). In contrast, a carotid US performed in patients with RR > 1 who had CRP levels greater than 3 mg/L at time of disease diagnosis yielded a sensitivity and a specificity of 60% and 77.4%, respectively (AUC: 0.69) (model 2). Interestingly, the sensitivity of the model was higher (75%) if the carotid US was performed in young AS patients with RR > 1 regardless of CRP data at the time of disease diagnosis but the specificity decreased to 56.6% (AUC: 0.66) (model 3) (Table 3).

## 4. Discussion

This is the first study aimed at evaluating the ability of a RR chart score to improve the identification of high risk CV disease among young AS patients. In our study, the RR demonstrated being superior to the SCORE risk function to identify high CV risk AS patients under 50 years, thus allowing them to benefit from an intensive preventive therapy. This is a crucial aspect considering that statins have been shown to decrease mortality by 37% in AS, a figure that doubles that observed in the general population [12].

We also confirmed the limitations attributed to the SCORE system when applied to AS patients under 50 years [3]. Although 27% of our patients had severe carotid US findings, most of them had been classified as having “low risk” based on the SCORE algorithm. The use of RR improved the identification of high CV risk young AS patients. In this regard, AS patients with RR > 1 were almost three times more likely to have subclinical atherosclerosis than those with RR = 1 (39.5% versus 14.3%).

CRP at disease diagnosis, which probably mirrors the presence of a prolonged subclinical inflammatory burden since the diagnosis is often delayed in patients with AS, was also found to be useful to improve the identification of high risk CV disease AS patients. In keeping with that, the 2016 ESC guidelines consider CRP as a proatherogenic factor with a similar influence to that attributed to the traditional CV risk factors, also capable of improving CV risk estimation [3]. Several studies indicate the cut-off point at which CRP is associated with an increased risk of CV events at 3 mg/L [11, 13]. In our series, 52,1% of AS patients had CRP levels higher than 3 mg/L at time of disease diagnosis, before receiving anti-TNF drugs that may reduce their levels, and this value was significantly associated with the presence of carotid plaques even after adjustment for confounding factors. The combination of a RR > 1 plus a CRP level at disease diagnosis higher than 3 mg/L increased the probability of disclosing severe subclinical disease expressed by the presence of carotid plaques up to 60%. Using this predictive model, we would narrow the number of patients to be assessed by carotid US to only a third of the whole population of young AS. This model showed a lower sensitivity than that obtained by performing carotid US to all young AS patients with a RR chart score > 1. Nevertheless, it exhibited a significantly higher overall specificity resulting in a higher AUC value (0.69 versus 0.66). In addition, its feasibility in clinical practice was also superior.

Identification of high CV disease risk AS patients is an issue of major relevance to initiate prompt primary prevention and consequently reduce the potential risk of fatal CV events in these patients. Statins achieve a reduction in the number of major vascular events in the lowest risk categories at least as big as in the higher risk groups, according to a recent meta-analysis of clinical trials [14]. Another argument that supports this strategy is the fact that the use of statins in individuals with CRP higher than 2 mg/L has demonstrated decreasing mortality by more than 30%, regardless of the lipid level [15].

In conclusion, our study confirms that AS patients under 50 years often have subclinical atherosclerotic disease. Although they are not identified by the use of the SCORE algorithm, due to the presence of carotid plaques, these patients would be considered as having high CV risk. Nevertheless, RR chart score assessment may help to identify young AS patients at high risk of CV disease. Most of them may be identified by combining the use of a RR chart score along with CRP levels at disease diagnosis and carotid US, which are tools proposed by the 2016 ESC guidelines experts to improve CV disease risk stratification.

## Figures and Tables

**Table 1:** Main epidemiologic, clinical, and ultrasonography features of a series of 73 ankylosing spondylitis patients without cardiovascular events, diabetes mellitus, or chronic kidney disease between 35 and 50 years.

Variable	AS (*n* = 73)
Men/women, *n*	44/29
Age at the time of study (years), mean ± SD	41.49 ± 3.85
Age at the time of diagnosis (years), mean ± SD	35.84 ± 6.49
Delay to disease diagnosis (years), mean ± SD	7.59 ± 7.90
Disease duration (years), median (IQR)	
Since first symptoms	12.00 (7.00–19.50)
Since diagnosis of AS	5.00 (1.00–9.00)
BASDAI, mean ± SD	3.46 ± 2.07
ASDAS, mean ± SD	2.18 ± 0.91
BASFI, mean ± SD	3.31 ± 2.45
BASMI, mean ± SD	2.46 ± 1.30
MASES, median (IQR)	1.00 (0.00–4.00)
Extra-articular manifestations, *n* (%)	21 (28.77)
Psoriasis, *n* (%)	8 (10.96)
Inflammatory bowel disease, *n* (%)	3 (4.11)
Uveitis, *n* (%)	14 (19.18)
History of synovitis or enthesitis, *n* (%)	36 (49.32)
Syndesmophytes, *n* (%)	22 (30.14)
Therapy, *n* (%)	
Anti-TNF	32 (43.84)
DMARDs	35 (47.95)
NSAIDs	66 (90.41)
Corticosteroids	15 (20.55)
HLA-B27 positive, *n* (%)	55 (75.34)
CRP (mg/l), median (IQR)	
At time of study	2.00 (0.50–6.00)
At time of disease diagnosis	4.00 (2.00–10.50)
CRP > 3 mg/L at time of disease diagnosis, *n* (%)	38 (52.05)
ESR (mm/1st hour), median (IQR)	
At time of study	6.00 (3.00–14.50)
At time of disease diagnosis	10.00 (4.50–17.50)
History of classic cardiovascular risk factors, *n* (%)	
Current smokers	22 (30.14)
Have ever smoked	14 (19.18)
Obesity	15 (20.55)
Dyslipidemia	14 (19.18)
Hypertension	3 (4.11)
Blood pressure (mm Hg), mean ± SD	
Systolic	127.00 ± 13.84
Diastolic	79.47 ± 9.86
Cholesterol or triglycerides (mg/dl), mean ± SD	
Total cholesterol	198.00 ± 33.80
HDL cholesterol	54.57 ± 12.99
LDL cholesterol	122.40 ± 33.32
Triglycerides	87.82 ± 43.11
Carotid plaques, *n* (%)	20 (27.40)
SCORE-TC, *n* (%)	
Low (<1%)	59 (80.82%)
Moderate (≥1% and <5%)	14 (19.17%)
High (≥5% and <10%)	0
Very high (≥10%)	0

SD: standard deviation. IQR: interquartile range.

**Table 2:** Presence of carotid plaques in 73 AS patients between 35 and 50 years without cardiovascular events, diabetes mellitus, or chronic kidney disease who were categorized according their relative risk (RR) chart score.

RR		Carotid ultrasound
		Presence of carotid plaques
		*n* = 20 (27.4%)
1	*n* = 35 (47.9%)	5 (14.3%)
2	*n* = 22 (30.1%)	8 (36.4%)
3	*n* = 12 (16.4%)	5 (41.7%)
4	*n* = 3 (4.1%)	1 (33.3%)
5	*n* = 1 (1.4%)	1 (100%)
>1	*n* = 38 (52.1%)	15 (39.5%)

**Table 3:** Study of 73 AS patients between 35 and 50 years without cardiovascular events, diabetes mellitus, or chronic kidney disease. Sensitivity, specificity, percentage of correctly classified patients, and area under the ROC curve of three different models based on the TC-SCORE algorithm, the relative risk (RR) chart score, and a value of CRP higher than 3 mg/L at time of disease diagnosis along with the performance of carotid (US) to establish the presence of high/very high cardiovascular risk.

	Sensitivity	Specificity	Correctly classified	ROC [95% CI]
*Model 1* TC-SCORE ≥ 5% OR TC-SCORE ≥ 1% <5% plus carotid Carotid US (presence of plaques)	40%	88.7%	75.3%	0.64 [0.53–0.76]

*Model 2* RR > 1 and CRP > 3 mg/L at time of disease diagnosis plus carotid US (presence of plaques)	60.0%	77.4%	72.6%	0.69 [0.56–0.81]

*Model 3* RR > 1 plus carotid US (presence of plaques)	75.0%	56.6%	61.6%	0.66 [0.53–0.78]

TC-SCORE: total cholesterol systematic coronary risk evaluation, US: ultrasound, RR: relative risk, and CRP: C-reactive protein. The gold standard used to define high/very high cardiovascular risk was the presence of TC-SCORE ≥ 5% or carotid plaques.
